# Associations of inflammatory markers with post-acute clinical findings among survivors of Ebola virus disease with and without viral RNA shedding in the semen in Liberia: a nested case–control study

**DOI:** 10.1016/j.lanmic.2024.101033

**Published:** 2025-03-10

**Authors:** Mosoka P Fallah, Collin Van Ryn, J Soka Moses, Moses Badio, Tamba Fayiah, Kumblytee Johnson, Dehkontee Gayedyu-Dennis, Allen O Eghrari, Sheri D Weiser, Travis C Porco, Jeffrey N Martin, Michael J Peluso, David R McIlwain, Bonnie Dighero-Kemp, Elizabeth Higgs, Lisa E Hensley, George W Rutherford, Cavan Reilly, J Daniel Kelly

**Affiliations:** Partnership for Research on Vaccines and Infectious Diseases in Liberia (PREVAIL), Monrovia, Liberia; Africa Centres for Disease Control and Prevention (Africa CDC), Addis Ababa, Ethiopia; Division of Biostatistics and Health Data Science, University of Minnesota, Minneapolis, MN, USA; Partnership for Research on Vaccines and Infectious Diseases in Liberia (PREVAIL), Monrovia, Liberia; Partnership for Research on Vaccines and Infectious Diseases in Liberia (PREVAIL), Monrovia, Liberia; Department of Epidemiology and Biostatistics, University of California, San Francisco (UCSF), San Francisco, CA, USA; Partnership for Research on Vaccines and Infectious Diseases in Liberia (PREVAIL), Monrovia, Liberia; Partnership for Research on Vaccines and Infectious Diseases in Liberia (PREVAIL), Monrovia, Liberia; Partnership for Research on Vaccines and Infectious Diseases in Liberia (PREVAIL), Monrovia, Liberia; The Johns Hopkins University School of Medicine, Baltimore, MD, USA; Department of Medicine, University of California, San Francisco (UCSF), San Francisco, CA, USA; Department of Epidemiology and Biostatistics, University of California, San Francisco (UCSF), San Francisco, CA, USA; F.I. Proctor Foundation, University of California, San Francisco (UCSF), San Francisco, CA, USA; Department of Epidemiology and Biostatistics, University of California, San Francisco (UCSF), San Francisco, CA, USA; Department of Medicine, University of California, San Francisco (UCSF), San Francisco, CA, USA; Department of Microbiology and Immunology, Stanford University, Stanford, CA, USA; Integrated Research Facility, National Institute of Allergy and Infectious Diseases, Fort Detrick, MD, USA; Division of Clinical Research, National Institute of Allergy and Infectious Diseases, Bethesda, MD, USA; Integrated Research Facility, National Institute of Allergy and Infectious Diseases, Fort Detrick, MD, USA; Department of Epidemiology and Biostatistics, University of California, San Francisco (UCSF), San Francisco, CA, USA; Institute for Global Health Sciences, University of California, San Francisco (UCSF), San Francisco, CA, USA; Division of Biostatistics and Health Data Science, University of Minnesota, Minneapolis, MN, USA; Department of Epidemiology and Biostatistics, University of California, San Francisco (UCSF), San Francisco, CA, USA; Department of Medicine, University of California, San Francisco (UCSF), San Francisco, CA, USA; F.I. Proctor Foundation, University of California, San Francisco (UCSF), San Francisco, CA, USA; Institute for Global Health Sciences, University of California, San Francisco (UCSF), San Francisco, CA, USA

## Abstract

**Background:**

A high proportion of survivors of Ebola virus disease (EVD) have post-acute sequelae of EVD (PASE), but the relationship between inflammation and PASE pathogenesis is poorly understood. This study tests the hypothesis that inflammation is associated with PASE among survivors with and without viral RNA shedding in the semen.

**Methods:**

This was a case–control study nested in a longitudinal cohort that recruited confirmed survivors of EVD and their uninfected contacts from the 2013–16 EVD epidemic in Liberia, starting on June 1, 2015. We included participants aged at least 18 years with clinical data and plasma available at cohort baseline for analysis. A semen donation substudy tested male survivors for Ebola virus RNA shedding in the semen. A sex-stratified and survivor-stratified random sample of cases (survivors) and controls (contacts) was obtained to select stored baseline plasma samples for cytokine testing of markers of inflammation, immune regulation, and antiviral responses. Serostatus of cases and controls was confirmed by Filovirus Animal Nonclinical Group assay. We identified inflammatory markers (adjusted p≤0·05) elevated in cases compared with controls and then used these biomarkers in analyses comparing survivors with and without pre-specified PASE-associated clinical findings (self-reported symptoms and abnormal examination findings). Survivors with viral RNA shedding in the semen formed subgroup analyses.

**Findings:**

Our analysis cohort consisted of 1044 participants (594 survivors of EVD and 450 uninfected contacts); 515 (49·3%) were female and 529 (50·7%) were male. The subcohort of 243 male survivors with data on viral shedding included 81 (33%) participants with viral shedding in semen. Median time from acute EVD to baseline was 317 days (IQR 271–366). Survivors of EVD showed a pattern of elevated inflammatory markers indicative of macrophage (MCP-1, IL-1β, and M-CSF) and angiogenic factor activation (VEGF-A) compared with controls (adjusted p<0·05). In survivors with viral shedding in the semen compared with controls, VEGF-A was the only inflammatory marker that was significantly higher (adjusted p<0·001). After restricting the analysis to survivors, each inflammatory marker had a specific pattern of clinical findings. Higher levels of IL-1β were associated with higher odds of urinary frequency (p=0·002), musculoskeletal abnormalities (p=0·003), and abdominal abnormalities (p=0·03). By contrast, higher levels of MCP-1 were associated with lower odds of the same clinical findings. M-CSF was the only inflammatory marker associated with lower odds of joint pain (p=0·04). Higher levels of VEGF-A were associated with higher odds of abnormal chest findings in the overall survivor group (p=0·02) and in the subgroup with viral shedding in the semen (p=0·02).

**Interpretation:**

We found evidence of distinct biological pathways for PASE. Although viral RNA shedding in the semen could be associated with angiogenic activation, it did not explain many of the PASE symptoms and exam findings associated with the elevated macrophage markers, suggesting the pathobiology of some clinical manifestations might be autoimmunity, immune dysregulation, or another biological mechanism. These findings could inform shared biological pathways with other infection-associated chronic conditions, including post-acute sequelae of SARS-CoV-2 infection.

**Funding:**

National Cancer Institute and National Institute of Allergy and Infectious Diseases at the US National Institutes of Health.

## Introduction

A sizable proportion of individuals who survive acute Ebola virus disease (EVD) have post-acute sequelae of EVD (PASE), including joint pain, memory loss, and viral shedding in the semen.^1,2^ Although Ebola virus clears from the blood relatively quickly,^3^ viral RNA persistence (referred to as viral persistence throughout) might continue in sites with immune privilege such as the eyes, CNS, and male reproductive tract,^4–6^ and could be the primary driver of inflammation, autoimmunity, and other potential biological mechanisms of PASE. The pathogenesis of uveitis (ie, ocular inflammation) is the most well studied example of PASE and probably occurs due to multiple inter-related biological mechanisms such as a direct cytopathic effect, chronic systemic inflammation, viral persistence, and autoimmunity. These biological mechanisms could explain the pathogenesis of other PASE, including symptoms (eg, joint pain) and physical examination findings (eg, musculoskeletal abnormalities).^7^

Although there is a body of literature documenting viral RNA shedding (referred to as viral shedding throughout) in the semen and its relationship to PASE (eg, eye and joint findings), the effect of viral shedding on inflammatory markers and the independent role of inflammatory markers with PASE are understudied. Acute and chronic inflammatory responses have been described among survivors who show complete recovery without clinical sequelae,^8^ but few studies have explored patterns of inflammation and immune activation in those with clinical sequelae.^3^ Early in acute EVD, infected individuals are reported to have a transient release of IL-1β, IL-6, TNFα, MIP-1α, and MIP-1β,^9^ followed by an uncomplicated, relatively short recovery phase (without clinical sequelae) during which IL-1 receptor antagonist (IL-1Ra), soluble receptors for TNFα (sTNF-R), and soluble receptors for IL-6 (sIL-6R) persist.^8^ In a small substudy of the PostEboGui cohort in Guinea, established in March, 2015, towards the end of the 2013–16 EVD outbreak in west Africa, previously identified and other inflammatory markers (elevated IL-8, TNFα, IL-1RA, and soluble CD40L and CCL5) were detected at higher levels among survivors than healthy controls up to 2 years after EVD.^6^ This study also correlated chronic inflammation with the presence of any post-acute symptoms,^6^ suggesting that immune dysregulation (eg, activation of chemo-attractant non-specific immune cells) might partially explain PASE. Such dysregulation in post-acute disease processes, specifically in the upregulation of macrophages,^10^ has been reported in other infectious diseases (eg, HIV treated with antiretroviral therapy and SARS-CoV-2).^11,12^

To deepen insights into PASE pathogenesis and inform the design and deployment of targeted treatments, there is a need to assess whether PASE observed at least 1 year after acute EVD are associated with chronic inflammation and whether ongoing viral shedding is a driver of inflammation. The Partnership for Research on Ebola Virus in Liberia (PREVAIL) recruited a cohort of survivors of EVD and their close contacts (ie, controls) in June, 2015, towards the end of the 2013–16 west Africa EVD epidemic, and identified a set of symptoms and physical examination findings that were more prevalent among survivors than contacts (PREVAIL III cohort).^1^ In this report, we characterise the cytokine levels of survivors of EVD and contacts to investigate the association of inflammatory markers with PASE among survivors with and without viral shedding at their baseline visit in the PREVAIL III cohort.

## Methods

### Study design and participants

This was a case–control study nested in a longitudinal cohort of survivors of EVD and contacts that started on June 1, 2015, and was implemented through a partnership between the Liberian Ministry of Health and the National Institute of Allergy and Infectious Diseases (NIAID) at the US National Institutes of Health.

In the longitudinal cohort, described in detail elsewhere,^1^ survivors of EVD from the 2013–16 epidemic in Liberia were enrolled approximately 12 months following acute EVD (study baseline) if they had a documented diagnosis of EVD, had been treated at an Ebola treatment unit, and were listed in the EVD registry created by the Liberian Ministry of Health. Survivors of EVD provided information regarding their close contacts while acutely ill or sexual contacts after recovery. Contacts with no history of EVD were invited to enrol as controls. Survivors of EVD and close contacts underwent similar study procedures following the same visit schedule (ie, symptom checklist, physical examination, and blood collection) at study baseline (median of 317 days [IQR 271–366] after acute EVD) and every 6 months thereafter, for up to 5 years. All survivors and their close contacts enrolled in the longitudinal cohort study were eligible for the ocular substudy. A subset of participants underwent detailed ocular examination by multiple trained ophthalmologists at which time the diagnosis of uveitis^1^ was made using the Standardization of the Uveitis Nomenclature.^13^ All male survivors of EVD were eligible for a substudy to donate semen samples for Ebola virus RNA testing, but only a subset participated in this substudy.

In this nested case–control study, we included participants aged at least 18 years at baseline with baseline plasma available for analysis. We included survivors of EVD who were confirmed to be seropositive by anti-glycoprotein Ebola virus IgG serological testing with Filovirus Animal Nonclinical Group (FANG) assay.^1,14,15^ Among contacts, we included those confirmed to be seronegative by the FANG immunoassay and with no history of EVD. As a result, cases were defined as seropositive survivors of EVD, and controls were defined as seronegative contacts. We drew a sex-stratified and survivor-stratified random sample of the parent cohort, using a computer-based random number generator, to identify cases and controls. We oversampled for survivors with uveitis and other reported sequelae as well as male survivors with viral RNA shedding. Oversampling of those with reported sequelae provided greater power than a strictly random sample. This study was designed to be an exploratory analysis that identified associations between markers and specific PASE, generating hypotheses for future research. The sampling approach from the parent cohort formed the various analyses (appendix p 2).

The National Research Ethics Board of Liberia, the University of California, the San Francisco Institutional Review Board, and the NIAID Institutional Review Board approved the study protocol at the National Institutes of Health. Written informed consent was obtained from all participants. This study is registered with ClinicalTrials.gov, NCT02431923 (completed).

### Procedures

Patient demographic data collected at baseline included age, self-reported gender (male and female options), and highest schooling completed (ie, no formal education, primary school, junior high school, high school, vocational school, or university). Our explanatory variables were baseline plasma cytokine levels. At the AIDS Monitoring Laboratory within the Frederick National Laboratory for Cancer Research (Frederick, MD, USA), we used the Ella Automated Immunoassay System by ProteinSimple, part of Bio-Techne (Minneapolis, MN, USA), to measure levels of the following cytokines from stored plasma samples: CRP, IFN-β, IL-1Ra, IL-1α and IL-1β, IL-6, TNFα, TNF-R1, TNF-R2, ICAM-1, MCP-1, MCP-2, M-CSF, CD14, granzyme A and granzyme B, IL-10, IL-5, IL-8, IL-2, IL-2 receptor antagonist (IL-2Ra), MIP-1α, MIP-1β, and VEGF-A and VEGF-B.

This cytokine panel was designed in 2019 before any studies assessing chronic inflammation among survivors of EVD. Given the lack of an evidence base, the cytokines in this panel were selected because of their crucial role in inflammation, immune regulation, and antiviral responses from chronic inflammatory conditions. Many of these cytokines (eg, CRP, IL-6, TNF-R1, TNF-R2, IL-1β, MCP-1, and sCD14) were identified from the HIV literature that linked persistent inflammation with increased morbidity and mortality.^16–18^ Using the broader non-HIV evidence base, we also considered cytokines potentially linked to major post-acute sequelae such as uveitis, viral RNA shedding, and VEGF-A.^19,20^

### Outcomes

Outcomes included the following baseline pre-specified PASE-associated self-reported symptoms and physical examination abnormalities: fatigue, headache, muscle pain, joint pain, urinary frequency, memory loss, abdominal findings, musculoskeletal findings, neurological findings, and uveitis (acute and inactive). To reduce the likelihood of type 1 errors, we examined previously reported clinical findings associated with post-EVD clinical sequelae, as reported in the 1-year assessment of PREVAIL III.^1^ We defined these clinical findings as evidence of probable PASE.

We also assessed viral RNA shedding in the semen. We used the modified GeneXpert Ebola (Cepheid, Sunnyvale, CA) RT-PCR to target the nucleoprotein and glycoprotein regions of viral RNA present in seminal fluid.^21^ Male survivors were asked to provide specimens at baseline and every 2 weeks over the follow-up period. We assumed that male survivors had viral persistence if they had at least one positive semen specimen during the 1–2 years following EVD. Thus, we classified any male survivors with at least one positive semen test as a shedder.

### Statistical analysis

This study had two phases of statistical analysis. In phase 1, we measured the association of being a seropositive survivor of EVD (having had acute EVD) versus a seronegative contact (not having had acute EVD) with post-acute inflammatory markers at study baseline. After obtaining the cytokine data and identifying the cytokines that were significantly higher among survivors than controls, we were able to proceed with phase 2 and assess the association of inflammatory markers at baseline with clinical findings (symptoms and examination abnormalities) at baseline among survivors only.

Cytokine levels were log_2_-transformed and treated as continuous predictor variables unless more than 10% of participants had a cytokine level below the limit of detection. Then, these cytokines were treated as dichotomous predictor variables. Clinical findings (symptoms and physical examination abnormalities) and viral RNA shedding were dichotomous outcomes. The prevalence of these clinical findings was reported for survivors and contacts. Given that other illnesses could have resulted in these clinical findings, we also reported the excess prevalence of these clinical findings. Excess prevalence was calculated by subtracting the prevalence of clinical findings reported among survivors from those reported among contacts.

We assessed associations of inflammatory markers by comparing survivors with contacts. We used logistic regression models incorporating generalised estimating equations with an independent working correlation structure to estimate odds ratios (ORs), adjusting for age, sex, survivor status, and clustering for survivor–contact relationships.

Given that 25 inflammatory markers were examined among survivors and contacts, we used the Holm procedure to control type 1 errors from multiple hypothesis testing. Following the Holm test, we considered adjusted p values of 0·05 to be the statistical significance cutoff when comparing inflammatory markers among survivors (overall and subgroups) with contacts. Markers that differed significantly between survivors and contacts based on adjusted p values were tested for associations with sequelae among survivors.

We then restricted the cohort to survivors and compared survivors with and without the outcome under investigation. For each PASE-associated clinical finding (eight self-reported symptoms and physical examination abnormalities), we employed a logistic regression model incorporating generalised estimating equations to estimate ORs, adjusting for age and sex. We considered p-values of 0·05 or less to be statistically significant.

In addition, we conducted exploratory analyses with inflammatory markers identified from comparing survivors with contacts using an unadjusted p value of 0·01 or less. Given the biological hypotheses of a relationship between IL-6 and clinical sequelae from HIV and COVID-19,^22^ and intermittent viral shedding and some inflammatory markers such as MCP-1, these relationships were also explored.

We repeated these analyses with male survivors, using the presence of intermittent viral shedding as a subgroup of interest. We did not impute missing data. All statistical analyses were performed with R, version 3.2.3.

### Role of the funding source

The NIAID, but not the National Cancer Institute, contributed to the design and conduct of the study. These sponsors did not have a role in the management, analysis, and interpretation of the data; the preparation, review, or approval of the manuscript; or the decision to submit the manuscript for publication.

## Results

Based on our sampling strategy for the 2258 participants in the parent cohort (760 seropositive survivors and 1498 seronegative contacts), we formed an analytic cohort of 1044 overall participants, comprising 594 (57%) seropositive survivors of EVD and 450 (43%) seronegative contacts (controls; appendix p 1). Table 1 presents complete demographic results of the analytic cohort.

In the group of 594 survivors (table 1), 307 (51·7%) participants were male and 287 (48·3%) were female. Of the 307 male survivors, 243 (79·2%) had available data on viral shedding in the semen. Ophthalmic data were available for 1041 (99·7%) of 1044 overall participants. The median time from acute EVD onset to baseline study visit was 317 days (IQR 271–366). Most inflammatory markers measured at baseline reached detectable levels among all or nearly all participants and subgroups. Cytokine levels were below the limit of detection in more than 10% of participants for the following inflammatory markers: IL-1α, IL-1β, IL-2, IL-5, IL-6, and INF-β (appendix pp 3–5). Among the 243 men sampled for RNA viral shedding in semen, 81 (33·3%) had viral shedding.

Table 2 presents the prevalence of symptoms and physical examination findings reported among survivors and controls. Excess prevalence of at least one PASE-associated clinical finding was 18·8% (538 [90·6%] of 594 survivors minus 323 [71·8%] of 450 controls). Joint pain (excess prevalence 24·0%; 325 [54·7%] of 594 survivors minus 138 [30·7%] of 450 controls) and memory loss (22·4%; 185 [31·1%] of 594 survivors minus 39 [8·7%] of 450 controls) were among the PASE-associated symptoms with the highest excess prevalence. Uveitis (12·8%; 207 [35·0%] of 591 survivors minus 100 [22·2%] of 450 controls) was the PASE-associated physical examination finding with the highest excess prevalence. Other than uveitis, which occurred more in the group of survivors with viral shedding in the semen than survivors without shedding, the distribution of clinical findings was similar when comparing all survivors with the subgroup of survivors with viral shedding in the semen.

Compared with controls, survivors of EVD had higher levels of MCP-1 (adjusted p=0·02), IL-1β (adjusted p=0·002), M-CSF (adjusted p=0·02), and VEGF-A (adjusted p<0·001; table 3, figure). Other inflammatory markers were not higher in survivors than controls.

In subgroup analyses, VEGF-A was the only inflammatory marker found to be higher among survivors with viral shedding in the semen compared with controls (adjusted p<0·001). Of the other 24 inflammatory markers evaluated, none were higher among survivors with viral shedding compared with controls (table 3).

In analyses of survivors with and without PASE-associated clinical findings, we found that MCP-1, M-CSF, and VEGF-A had a different direction of association with clinical findings compared with IL-1β. Higher levels of MCP-1, M-CSF, and VEGF-A were associated with lower odds of clinical findings whereas higher levels of IL-1β were associated with higher odds of clinical findings (table 4).

Each inflammatory marker had a specific pattern of clinical findings. Although IL-1β and MCP-1 had opposing directions of association, these biomarkers were associated with a similar distribution of clinical findings. Higher levels of IL-1β were associated with higher odds of urinary frequency (p=0·002), musculoskeletal abnormalities (p=0·003), and abdominal abnormalities (p=0·03). By contrast, higher levels of MCP-1 were associated with lower odds of the same clinical findings (p=0·03 for urinary frequency; p=0·01 for musculoskeletal abnormalities; and p<0·001 for abdominal abnormalities). M-CSF was the only inflammatory marker associated with lower odds of joint pain (p=0·04).

Several of these clinical findings were associated with more than one biomarker. Specifically, urinary frequency, musculoskeletal abnormalities (muscle tenderness, decreased joint range of motion, and joint swelling), and abdominal abnormalities (tenderness, mass, and distension) were associated with more than one biomarker. Compared with survivors who did not have abdominal abnormalities, for example, higher levels of MCP-1 (p<0·001), VEGF-A (p=0·01), and IL-1β (p=0·03) were associated with the odds of abdominal abnormalities.

We found an association between higher levels of VEGF-A and abnormal chest findings such as irregular pulse, decreased breath sounds, and heart murmur in the overall survivor group (p=0·02; adjusted OR 1·40) and in the subgroup with viral shedding in the semen (p=0·02; adjusted OR 3·38; table 5).

In unadjusted but not adjusted analyses, MIP-1β had higher levels in survivors than in controls, so we decided to explore the relationship between higher levels of MIP-1β and clinical findings. Higher levels of MIP-1 beta were associated with higher odds of headache, although this was not statistically significant (p=0·06; appendix p 5).

We also explored IL-6 among the survivors of EVD and the subgroup with viral shedding in the semen because IL-6 is associated with post-acute disease pathology among those with HIV (appendix p 6).^22^ In comparisons of survivors with controls, higher levels of IL-6 were associated with higher odds of any sequelae (p=0·04) and lower odds of neurological abnormalities such as abnormal reflexes, tremor, and cranial nerve abnormalities (p=0·01). Among survivors with viral shedding in the semen, higher levels of IL-6 were associated with muscle pain (p=0·01).

## Discussion

In this large cohort of survivors of EVD and controls (ie, seronegative contacts), survivors had evidence of higher levels of MCP-1, IL-1β, M-CSF, and VEGF-A. These elevated inflammatory markers were associated with several PASE-associated clinical findings, including urinary frequency, joint pain, and musculoskeletal, chest, and abdominal abnormalities. We observed distinct, elevated cytokine patterns among survivors with viral shedding (VEGF-A) and without viral shedding in the semen (MCP-1, IL-1β, and M-CSF). Although angiogenic activation (VEGF-A) and chest abnormalities were found in the male survivors with viral shedding in the semen, viral shedding did not explain many of the PASE symptoms and physical examination findings associated with the elevated macrophage markers (MCP-1, IL-1β, and M-CSF) found in the overall survivor cohort, suggesting autoimmunity, immune dysregulation, or other biological mechanisms might be causing some clinical manifestations.^6,23^ These biological mechanisms probably represent only two pathways in a more complex process of PASE pathogenesis.

Although there have been conflicting findings about chronic inflammation in survivors,^24^ our study supported immune activation among survivors. These findings build on previous work by Wiedemann and colleagues on a small EVD cohort in Guinea, which identified chronic inflammation among survivors.^6^ In contrast with other survivor cohorts, our cohort includes a subgroup of survivors with viral shedding inthe semen. We found that viral shedding in the semen was associated with higher levels of VEGF-A, promoting a breakdown of the blood–testes barrier, and potentially allowing increased access to such immune-privileged sites by the immune system.^25^ Although real-world sexual transmission of Ebola virus has been rarely reported,^26^ persistence of viral RNA in the semen has been more common due to several antiviral defence mechanisms in humans that facilitate survival of cells infected with viral RNA as an unintended consequence of suppressing the production of infectious virions.^27^ Given that Ebola virus can infect various cell types (eg, endothelial cells and macrophages), mechanistic study of post-acute EVD pathogenesis could elucidate which cell type serves as the reservoir of the virus and the origin of VEGF-A.

Whereas a previous small study was underpowered to detect a link between inflammatory markers and specific clinical sequelae,^6^ our large study showed patterns of clinical findings such as the different directions of association with higher levels of MCP-1 and IL-1β. Given that higher levels of MCP-1 were associated with lower odds of specific clinical sequelae, one interpretation of the negative association could be downregulation of the biomarker, suggesting that survivors with these clinical findings were no longer replicating or shedding the RNA virus. More than one timepoint would be needed to confirm this interpretation of findings, but MCP-1 is part of a biological pathway involving monocyte and macrophage activation that might have been triggered by Ebola virus replication.^28^ We also know from other studies published on this cohort that many of these survivors were in the process of recovering from these clinical findings.^1^ Taken together, our findings and other studies of this cohort support the hypothesis that higher levels of MCP-1 could be a predictor of recovery among survivors. Another interpretation of these partly explained relationships supports a hypothesis that differing biological mechanisms such as immune dysregulation instead of viral persistence contribute to different clinical phenotypes (eg, linking immune dysregulation to a musculoskeletal phenotype).^7^ In particular, pathways involving monocyte and macrophage activation have become a focus in treated HIV infection in the past 5 years, a condition in which this immune activation is associated with atherosclerotic inflammation and for which IL-1β targeted therapies have been proposed.^17,29,30^

We recognise several limitations. First, this was a nested case–control study, so we can identify associations between inflammatory markers and PASE-associated clinical findings, but we cannot deduce causation. Second, this study was conducted in a resource-limited setting, where accurate medical histories do not exist in medical records nor by self-report. Covariates such as accurate medical histories and the severity of acute illness were not measured, representing potential confounders that could have resulted in some bias. Third, most survivors were enrolled in this cohort nearly 1 year after acute illness, at which time illnesses other than EVD could have caused a proportion of the reported post-acute clinical findings. To mitigate potential measurement bias, we selected clinical findings known to be differentially more prevalent among survivors than contacts and reported excess prevalence. We also identified cytokines elevated among survivors compared with contacts. When examining the association, we expected that measurement error in the outcome would result in some degree of imprecision and type 2 errors. Fourth, our subgroup of male survivors of EVD with viral RNA shedding in the semen was small, and analyses with clinical findings were underpowered to detect small effects. Fifth, the large number of participants in this study required the use of a high-throughput assay that included 25 cytokines thought to represent major markers of inflammation, immune activation, and antiviral responses. However, these 25 cytokines represent only a fraction of the potential immune markers that can be profiled with immune-related proteomic assays. Finally, although we performed multiple comparisons, we adjusted p values in our approach to identifying differences in inflammatory markers between survivors and contacts, so we are confident in those associations.

Taken together, we conclude that 1 year after surviving EVD, overall survivors, regardless of viral shedding in the semen, had higher levels of MCP-1, IL-1β, M-CSF, and VEGF-A than controls. These inflammatory markers were associated with specific symptoms and physical examination abnormalities. Varying inflammatory marker signatures of PASE among survivors of EVD with and without viral shedding in the semen could represent distinct biological pathways of post-acute EVD pathogenesis. Higher levels of VEGF-A could serve as a biomarker for viral RNA persistence in the semen, whereas higher levels of MCP-1, IL-1β, and M-CSF might indicate the presence of another underlying biological mechanism, such as autoimmunity or immune dysregulation. The adverse downstream effects of these inflammatory markers on PASE-associated clinical findings should be considered in future studies elucidating biological targets of therapeutic PASE interventions. The markers we identified could also be of interest for efforts to study shared biological pathways across various infection-associated chronic conditions, including post-acute sequelae of SARS-CoV-2, myalgic encephalomyelitis or chronic fatigue syndrome, and post-treatment Lyme disease, among others.

## Supplementary Material

Fallah_Lancet_Microbe_2025_supplementary

## Figures and Tables

**Figure:**
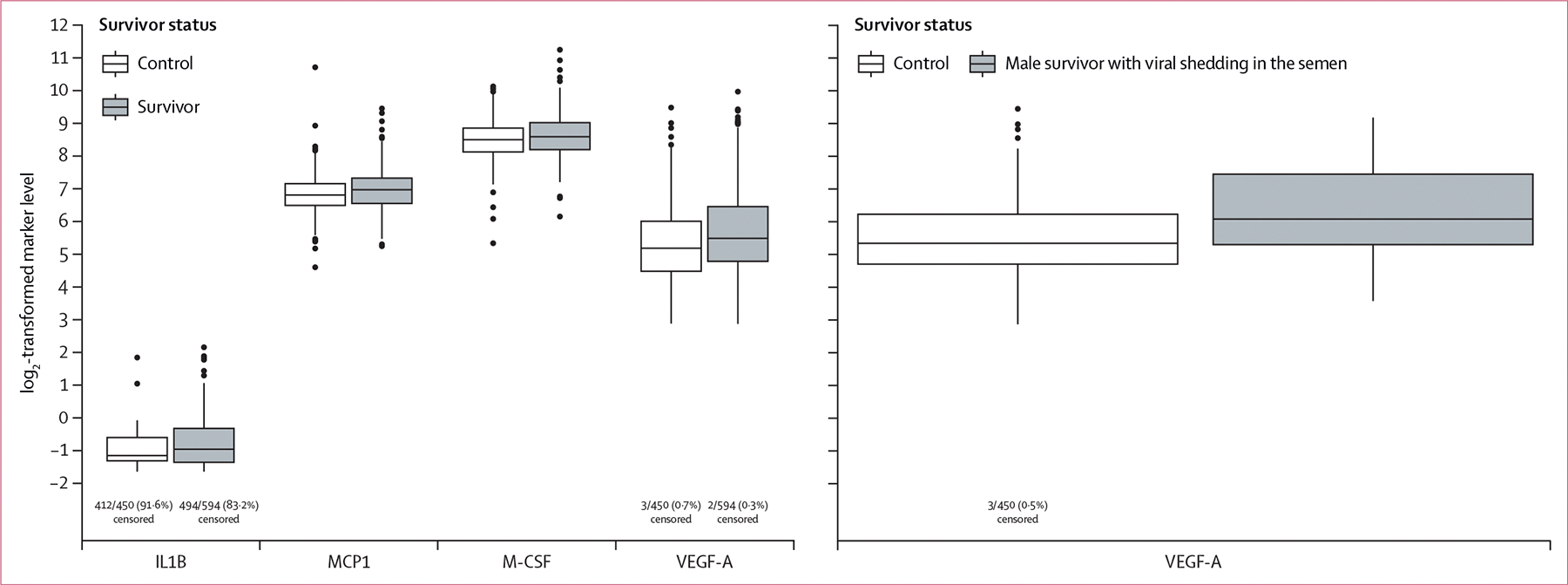
Inflammatory marker levels by survivor status for inflammatory markers that differ significantly between survivors of EVD (cases) and uninfected contacts (controls) based on adjusted p values Participants with a cytokine level below the LOD were censored; n/N (%) censored is provided for inflammatory markers with censored values. EVD=Ebola virus disease. LOD=limit of detection.

**Table 1:** Demographic summary of survivors of EVD (cases) and uninfected contacts (controls)

	Overall (N=1044)	Controls (N=450)	Survivors (N=594)

Sex			
Male	529/1044 (50·7%)	222/450 (49·3%)	307/594 (51·7%)
Female	515/1044 (49·3%)	228/450 (50·7%)	287/594 (48·3%)
Age at enrolment, years	33 (25–43)	33 (25–44)	33 (26–42)
Participants with ophthalmic data available	1041/1044 (99·7%)	450/450 (100%)	591/594 (99·5%)
Participants with viral shedding data available	..	..	243/594 (40·9%)

Data are n/N (%) or median (IQR). EVD=Ebola virus disease.

**Table 2:** Summary of clinical findings and viral shedding among uninfected contacts (controls), survivors of EVD (cases), and EVD survivors with viral shedding in the semen

	Controls (N=450)	Survivors (N=594)	Male survivors with viral shedding in semen (N=81)

Urinary frequency	20/450 (4·4%)	93/594 (15·7%)	12/81 (14·8%)
Fatigue	30/450 (6·7%)	112/594 (18·9%)	10/81 (12·3%)
Headache	195/450 (43·3%)	281/594 (47·3%)	26/81 (32·1%)
Muscle pain	54/450 (12·0%)	150/594 (25·3%)	13/81 (16·0%)
Joint pain	138/450 (30·7%)	325/594 (54·7%)	36/81 (44·4%)
Memory loss	39/450 (8·7%)	185/594 (31·1%)	16/81 (19·8%)
Uveitis	100/450 (22·2%)	207/591 (35·0%)	34/78 (43·6%)
Acute uveitis	12/450 (2·7%)	39/591 (6·6%)	5/78 (6·4%)
Musculoskeletal abnormalities	18/450 (4·0%)	47/594 (7·9%)	4/81 (4·9%)
Neurological abnormalities	13/450 (2·9%)	35/594 (5·9%)	6/81 (7·4%)
Chest abnormalities	17/450 (3·8%)	28/594 (4·7%)	4/81 (4·9%)
Abdominal abnormalities	49/450 (10·9%)	113/594 (19·0%)	5/81 (6·2%)
Any of the findings	323/450 (71·8%)	538/594 (90·6%)	81/81 (100%)

Data are n/N (%). EVD=Ebola virus disease.

**Table 3:** Associations of inflammatory markers with EVD survivor status

	Survivors (N=594)			Male survivors with viral shedding in semen (N=81)		
	Adjusted OR (95% CI)	p value	Corrected p value	Adjusted OR (95% CI)	p value	Corrected p value

**Macrophage-specific inflammatory markers**						
MCP-1	1·57 (1·21–2·05)	0·0008	0·019	1·96 (1·07–3·61)	0·031	0·61
MCP-2	1·22 (1·01–1·46)	0·036	0·60	1·39 (0·96–2·01)	0·084	1·00
TNF	1·26 (1·05–1·51)	0·012	0·24	1·07 (0·73–1·58)	0·73	1·00
TNF-R1	1·35 (0·99–1·84)	0·06	0·90	0·86 (0·45–1·63)	0·65	1·00
TNF-R2	1·20 (0·94–1·52)	0·14	1·00	0·83 (0·5–1·39)	0·48	1·00
MIP-1α	1·07 (0·98–1·16)	0·12	1·00	1·09 (0·92–1·28)	0·32	1·00
MIP-1β	1·27 (1·09–1·49)	0·0028	0·06	1·41 (0·88–2·24)	0·15	1·00
IL-1β	>10% censored	<0·0001*	0·0016	>10% censored	0·024*	0·52
IL-6	>10% censored	0·26*	1·00	>10% censored	1·00*	1·00
M-CSF	1·39 (1·14–1·69)	0·00099	0·022	1·36 (0·84–2·19)	0·21	1·00
**Non-macrophage-specific inflammatory markers**						
VEGF-A	1·28 (1·16–1·41)	<0·0001	<0·0001	1·63 (1·32–2·01)	<0·0001	<0·0001
VEGF-B	1·18 (0·95–1·47)	0·14	1·00	1·03 (0·63–1·70)	0·90	1·00
Granzyme A	1·19 (0·97–1·46)	0·099	1·00	1·54 (0·96–2·45)	0·071	1·00
Granzyme B	0·97 (0·87–1·08)	0·56	1·00	0·99 (0·82–1·20)	0·93	1·00
IL-10	1·03 (0·93–1·14)	0·57	1·00	0·84 (0·63–1·11)	0·22	1·00
IL-2Ra	1·02 (0·81–1·28)	0·87	1·00	0·79 (0·48–1·31)	0·36	1·00
IL-1Ra	1·04(0·88–1·23)	0·64	1·00	0·86 (0·50–1·47)	0·57	1·00
IL-8	1·11 (0·97–1·28)	0·13	1·00	1·47 (1·07–2·02)	0·016	0·37
ICAM-1	0·82 (0·64–1·04)	0·098	1·00	0·69 (0·46–1·03)	0·073	1·00
CD14	1·33 (1·03–1·72)	0·028	0·51	2·04 (1·17–3·55)	0·012	0·28
CRP	1·08 (1·01–1·15)	0·026	0·50	0·97 (0·85–1·11)	0·64	1·00
IL-1α	>10% censored	1·00*	1·00	>10% censored	1·00*	1·00
IL-2	>10% censored	0·41*	1·00	>10% censored	0·57*	1·00
IL-5	>10% censored	0·036*	0·60	>10% censored	0·024*	0·52
IFN-β	>10% censored	0·082*	1·00	>10% censored	0·58*	1·00

ORs are adjusted for age and sex and estimate the odds of having higher levels of an inflammatory marker among survivors (N=594) as compared with controls (N=450). Adjusted and unadjusted p values are shown. >10% censored means more than 10% of participants had a cytokine level below the LOD. EVD=Ebola virus disease. IL-1Ra=IL-1 receptor antagonist. IL-2Ra=IL-2 receptor antagonist. LOD=limit of detection. OR=odds ratio.

* Fisher’s exact test p value.

**Table 4:** Adjusted ORs by clinical finding among survivors of EVD for inflammatory markers

	Number of participants (% of 594 overall survivors)	MCP-1		IL-1β		VEGF-A		M-CSF	
		Adjusted OR (95% CI)	p value	Adjusted OR (95% CI)	p value	Adjusted OR (95% CI)	p value	Adjusted OR (95% CI)	p value

Urinary frequency	93 (15·7%)	0·62 (0·41–0·95)	0·029	2·438 (1·37–4·26)	0·002	1·00 (0·84–1·19)	0·98	0·88 (0·62–1·24)	0·47
Fatigue	112 (18·9%)	0·85 (0·58–1·24)	0·39	0·967 (0·53–1·7)	0·91	0·87 (0·74–1·02)	0·095	0·92 (0·67–1·27)	0·63
Headache	281 (47·3%)	1·00 (0·74–1·35)	1·00	1·019 (0·65–1·61)	0·93	0·96 (0·84–1·08)	0·48	0·79 (0·61–1·02)	0·071
Muscle pain	150 (25·3%)	1·02 (0·73–1·43)	0·90	1·225 (0·73–2·02)	0·43	0·87 (0·75–1·00)	0·055	0·84 (0·63–1·12)	0·23
Joint pain	325 (54·7%)	1·03 (0·76–1·39)	0·85	1·192 (0·76–1·88)	0·44	0·95 (0·84–1·07)	0·40	0·77 (0·59–0·99)	0·043
Memory loss	185 (31·1%)	0·66 (0·47–0·91)	0·012	1·392 (0·86–2·23)	0·17	0·94 (0·82–1·08)	0·37	0·78 (0·59–1·03)	0·077
Uveitis	207 (35%)	0·99 (0·73–1·36)	0·97	1·329 (0·83–2·11)	0·23	1·12 (0·98–1·27)	0·085	1·13 (0·87–1·48)	0·37
Acute uveitis	39 (6·6%)	0·96 (0·53–1·75)	0·90	1·137 (0·41–2·7)	0·79	1·06 (0·83–1·35)	0·66	0·92 (0·56–1·53)	0·76
Viral shedding in the semen	81 (33·3%)	1·74 (1·04–2·93)	0·035	0·821 (0·41–1·61)	0·57	1·52 (1·21–1·91)	<0.001	1·19 (0·74–1·9)	0·47
Musculoskeletal abnormalities	47 (7·9%)	0·47 (0·26–0·84)	0·01	3·041 (1·43–6·24)	0·0028	1·05 (0·84–1·32)	0·66	0·81 (0·51–1·29)	0·37
Neurological abnormalities	35 (5·9%)	0·85 (0·45–1·60)	0·61	1·28 (0·49–2·95)	0·58	0·76 (0·57–1·01)	0·056	0·67 (0·39–1·15)	0·15
Chest abnormalities	28 (4·7%)	1·01 (0·50–2·05)	0·98	0·779 (0·18–2·39)	0·70	1·40 (1·06–1·85)	0·019	0·61 (0·33–1·13)	0·11
Abdominal abnormalities	113 (19%)	0·35 (0·23–0·55)	<0·0001	1·964 (1·07–3·53)	0·025	0·79 (0·67–0·95)	0·011	0·97 (0·7–1·35)	0·87
Any ofthe findings	538 (90·6%)	0·85 (0·51–1·43)	0·54	1·423 (0·7–3·16)	0·36	0·96 (0·78–1·18)	0·68	1·09 (0·69–1·72)	0·72

ORs are adjusted for age and sex (except for viral shedding, which is adjusted for age and number of semen specimens collected) and estimate the odds of having higher levels of an inflammatory marker among survivors with a clinical finding as compared with survivors without the same clinical finding. The p-values shown here are not corrected for multiple comparisons. EVD=Ebola virus disease. OR=odds ratio.

**Table 5:** Adjusted ORs by clinical finding among survivors of EVD with viral shedding in the semen for VEGF-A, which differed significantly between survivors and controls based on adjusted p values

	Number of participants (% of 81 survivors with viral shedding in the semen)	VEGF-A	
		Adjusted OR (95% CI)	p value

Urinary frequency	12/81 (14·8%)	0·89 (0·54–1·43)	0·63
Fatigue	10/81 (12·3%)	0·72 (0·40–1·21)	0·23
Headache	26/81 (32·1%)	0·86 (0·59–1·23)	0·41
Muscle pain	13/81 (16·0%)	1·06 (0·67–1·68)	0·79
Joint pain	36/81 (44·4%)	1·27 (0·91–1·79)	0·17
Memory loss	16/81 (19·8%)	0·81 (0·52–1·22)	0·32
Uveitis	34/78 (43·6%)	1·33 (0·95–1·90)	0·10
Acute uveitis	5/78 (6·4%)	1·06 (0·53–2·09)	0·86
Musculoskeletal abnormalities	4/81 (4·9%)	1·04 (0·47–2·22)	0·93
Neurological abnormalities	6/81 (7·4%)	0·98 (0·51–1·82)	0·94
Chest abnormalities	4/81 (4·9%)	3·38 (1·35–11·81)	0·023
Abdominal abnormalities	5/81 (6·2%)	1·43 (0·73–3·00)	0·31
Any ofthe findings	81/81 (100·0%)	..	..

ORs are adjusted for age and number of semen specimens collected. ORs estimate the odds of having higher levels of VEGF-A among survivors with viral shedding in the semen who do have a clinical finding vs those who do not have this clinical finding. The p-values shown here are not corrected for multiple comparisons. EVD=Ebola virus disease. OR=odds ratio.

## Data Availability

De-identified participant data will be available on request to the principal investigator (MPF) with evidence of institutional review board approval from the National Research Ethics Board of Liberia (as stated in the study protocol).

## Research in context

### Evidence before this study

We did a systematic review of studies assessing the association of inflammatory markers with post-acute clinical sequelae of Ebola virus disease (PASE), inclusive of those with viral RNA shedding data. We searched PubMed and Web of Science using the search string “Ebola virus disease (EVD) AND clinical sequelae AND (viral persistence OR viral shedding OR inflammation)”, as well as reference lists and conference reports such as those from the Filovirus Animal Non-Clinical Group Ebola Workshop. We last updated the search on May 10, 2024, and used no language restrictions. Although we found a body of literature documenting the persistence of Ebola virus and its relationship to PASE-associated clinical findings, studies assessing inflammatory markers as part of a biological pathway to PASE-associated clinical findings are missing from the literature. Evaluations of inflammatory markers and PASE-associated clinical findings in the presence and absence of viral persistence or RNA shedding are also inadequate.

### Added value of this study

Evidence for chronic inflammation among survivors of EVD has varied depending on the cohortstudy, its sample size, the timing of data collection in the post-acute period, the presence of key subpopulations (eg, viral shedding subgroup), and the use of a control group. Although chronic inflammation among survivors of EVD has been shown, previous studies were underpowered to make strong links to PASE. This nested case–control study in Liberia followed the 2013–16 Ebola outbreak. Its added value was its large sample size, relatively early post-acute study visits, identification of male survivors of EVD with viral shedding in the semen, and use of a control group. As a result, this study was able to identify inflammatory markers elevated among cases (survivors) compared with controls (contacts) and subsequently use these biomarkers in analyses comparing survivors with and without pre-specified, PASE-associated clinical findings. The subgroup analyses among survivors with viral shedding in the semen were unique and allowed for the evaluation of two biological mechanisms in one study. Because of the nature of this cohort, we found evidence of distinct biological pathways of PASE. Namely, viral RNA shedding in the semen was associated with angiogenic activation but not macrophage activation, whereas macrophage activation was more strongly associated with PASE-associated clinical findings, particularly memory loss, urinary frequency, musculoskeletal abnormalities, and abdominal abnormalities.

### Implications of all the available evidence

This report highlights inflammatory markers as one of several biological mechanisms that might be associated with post-acute clinical findings and ultimately serve as biological targets for interventions designed to prevent and treat PASE. Evidence also supports viral RNA persistence as an important biological target. Autoimmunity and immune dysregulation could be additional biological mechanisms considered in future studies. The markers identified in this report might also be of interest for efforts to study shared biological pathways across various infection-associated chronic conditions, including post-acute sequelae of SARS-CoV-2, myalgic encephalomyelitis or chronic fatigue syndrome, and post-treatment Lyme disease, among others.
