# Supplementary material for: Associations of inflammatory markers with post-acute clinical findings among survivors of Ebola virus disease with and without viral RNA shedding in the semen in Liberia: a nested case–control study
Source: Lancet Microbe. Author manuscript; Available in PMC 2025 Sep 12. (PMC12425654; doi:10.1016/j.lanmic.2024.101033)
Supplement: Fallah_Lancet_Microbe_2025_supplementary [file NIHMS2107176-supplement-Fallah_Lancet_Microbe_2025_supplementary.pdf]

THE LANCET
Microbe

Supplementary appendix

This appendix formed part of the original submission and has been peer reviewed.
We post it as supplied by the authors.

Supplement to: Fallah MP, Van Ryn C, Moses JS, et al. Associations of inflammatory markers with post-acute clinical findings among survivors of Ebola virus disease with and without viral RNA shedding in the semen in Liberia: a nested case–control study. *Lancet Microbe* 2025. https://doi.org/10.1016/j.lanmic.2024.101033

Supplemental Material

Associations of inflammatory markers with post-acute clinical findings among survivors of Ebola virus disease with and without viral RNA shedding in the semen in Liberia: a nested case–control study

Mosoka P Fallah, Collin Van Ryn, J Soka Moses, Moses Badio, Tamba Fayiah, Kumblytee Johnson, Dehkontee Gayedyu-Dennis, Allen O Eghrari, Prof. Sheri D Weiser, Prof. Travis C Porco, Prof. Jeffrey N Martin, Michael J Peluso, David R McIlwain, Bonnie Dighero-Kemp, Elizabeth Higgs, Lisa E Hensley, Prof. George W Rutherford, Prof. Cavan Reilly, J Daniel Kelly

Partnership for Research on Vaccines and Infectious Diseases in Liberia (PREVAIL), Monrovia, Liberia (M P Fallah PhD, J S Moses, M Badio MS, T Fayiah BSc, K Johnson MD, D Gayedyu-Dennis MD)**; Africa Centres for Disease Control and Prevention (Africa CDC), Addis Ababa, Ethiopia** (M P Fallah)**; Division of Biostatistics and Health Data Science, University of Minnesota, Minneapolis, MN, USA** (C Van Ryn MS, C Reilly PhD)**; Department of Epidemiology and Biostatistics** (M Badio, T C Porco PhD, J N Martin MD, G W Rutherford MD, J D Kelly MD PhD)**, Department of Medicine** (J D Kelly, S D Weiser MD, M J Peluso MD)**, F.I. Proctor Foundation** (J D Kelly, T C Porco)**, and Institute for Global Health Sciences** (G W Rutherford, J D Kelly)**, University of California, San Francisco (UCSF), San Francisco, CA, USA; The Johns Hopkins University School of Medicine, Baltimore, MD, USA** (A O Eghrari MD)**; Department of Microbiology and Immunology, Stanford University, Stanford, CA, USA** (D R McIlwain PhD)**; Integrated Research Facility, National Institute of Allergy and Infectious Diseases, Fort Detrick, MD, USA** (B Dighero-Kemp BS, L E Hensley PhD)**; Division of Clinical Research, National Institute of Allergy and Infectious Diseases, Bethesda, MD, USA** (E Higgs MD).

Table of Contents

1. Figure S1 Flow Diagram of sex-stratified and survivor-stratified random sample of cases (survivors) and controls (contacts).
2. Table S1 Summary of inflammatory markers among EVD survivors and controls.
3. Table S2 Summary of inflammatory markers among EVD survivors.
4. Table S3 Summary of inflammatory markers among EVD survivors with viral shedding in the semen.
5. Table S4 Adjusted odds ratios by clinical finding for MIP-1B. The p-values shown here are not corrected for multiple comparisons. Odds ratios are adjusted for sex and age at enrollment (except for viral persistence which is adjusted for age and number of semen specimens collected) and refer to a two-fold increase in marker level.
6. Table S5 Adjusted odds ratios by finding for IL6. The p-values shown here are not adjusted for multiple comparisons. Odds ratios are adjusted for sex and age at enrollment (except for viral persistence which is adjusted for age and number of semen specimens collected) and refer to IL6 level being above (vs. below) the LOD.

Figure S1. Flow diagram of sex-stratified and survivor-stratified random sample of cases (survivors) and controls (contacts).

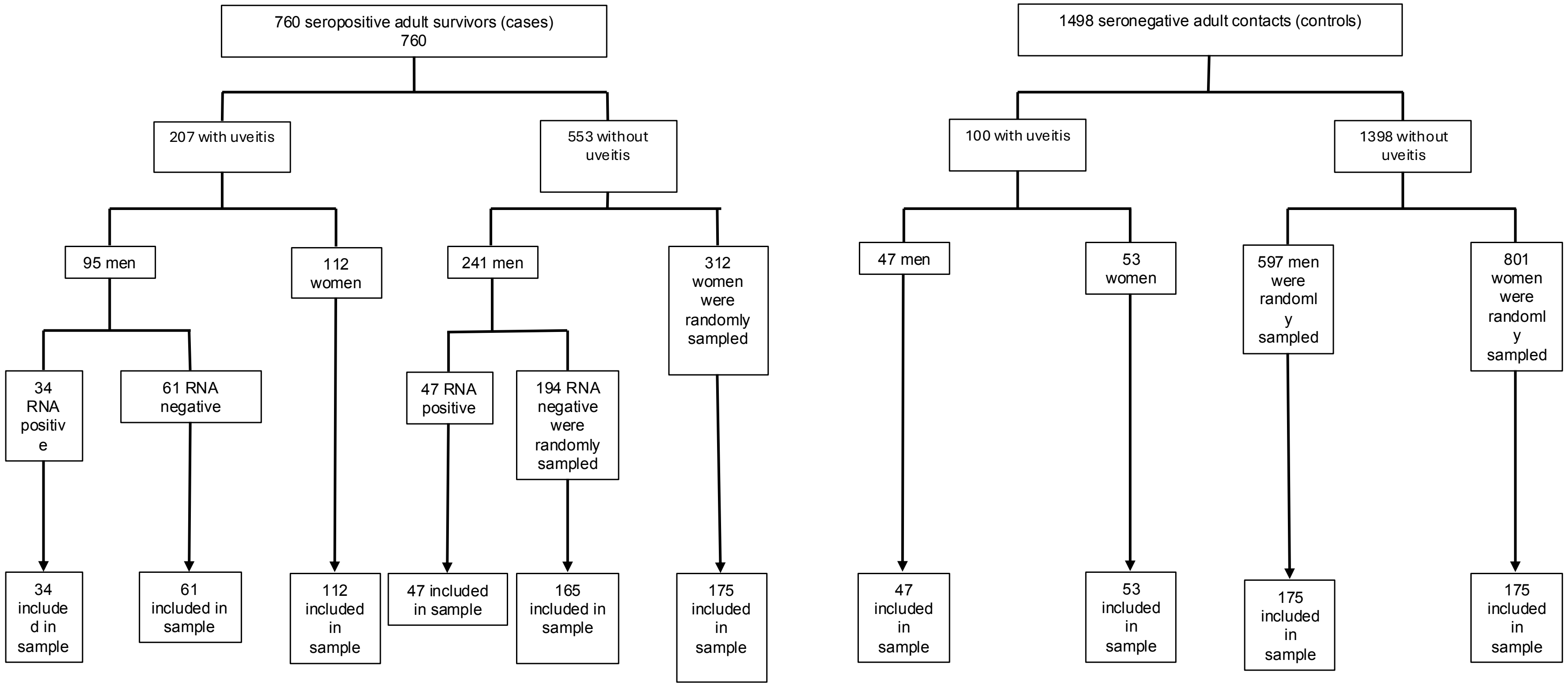

Table S1: Summary of inflammatory markers among EVD survivors and controls.

	N uncensored measurements	Med. (Q1, Q3)	log2-transformed Med. (Q1, Q3)	Coefficient of Variation of log2-transformed variable	N (%) below LOD	N (%) above LOD
Macrophage-specific inflammatory markers						
MCP1	1044	119.52 (91.78, 153.05)	6.9 (6.52, 7.26)	10.98	0 (0%)	0 (0%)
MCP2	1042	20.87 (15.88, 29.3)	4.38 (3.99, 4.87)	6.43	1 (0.1%)	0 (0%)
TNFA	1029	5.14 (4.01, 6.76)	2.36 (2, 2.76)	1.34	15 (1.4%)	0 (0%)
TNFR1	1043	912.91 (778.95, 1082.22)	9.83 (9.61, 10.08)	29.73	0 (0%)	0 (0%)
TNFR2	1043	2255.59 (1836.43, 2947.66)	11.14 (10.84, 11.53)	99.04	0 (0%)	0 (0%)
MIP1A	1037	27.26 (20.3, 40.35)	4.77 (4.34, 5.33)	58.11	0 (0%)	1 (0.1%)
MIP1B	1016	54.2 (40.2, 73.06)	5.76 (5.33, 6.19)	7.14	23 (2.2%)	0 (0%)
IL1B	138	0.1 (0.1, 0.1)	-3.25 (-3.25, -3.25)	-0.12	906 (86.8%)	0 (0%)
IL6	497	0.2 (0.2, 2.17)	-2.29 (-2.29, 1.11)	-7.55	546 (52.3%)	0 (0%)
MCSF	1043	375.11 (283.98, 491.98)	8.55 (8.15, 8.94)	23.78	0 (0%)	0 (0%)
Non-macrophage-specific inflammatory markers						
VEGFA	1039	40.15 (25.25, 77.73)	5.33 (4.66, 6.28)	18.05	5 (0.5%)	0 (0%)
VEGFB	1043	41.19 (32.34, 54.14)	5.36 (5.02, 5.76)	7.53	1 (0.1%)	0 (0%)
GRANA	1038	145.88 (114.87, 187.78)	7.19 (6.84, 7.55)	11.11	0 (0%)	0 (0%)
GRANB	1037	13.01 (8.77, 20.63)	3.7 (3.13, 4.37)	31.96	1 (0.1%)	0 (0%)
IL10	1040	5.54 (3.58, 10.41)	2.47 (1.84, 3.38)	6.38	4 (0.4%)	0 (0%)
IL2RA	1043	1426.39 (1155.14, 1816.52)	10.48 (10.17, 10.83)	65.8	0 (0%)	1 (0.1%)
IL1RA	1042	236.03 (181.76, 331.44)	7.88 (7.51, 8.37)	37.67	0 (0%)	0 (0%)
IL8	1036	5.07 (3.59, 7.42)	2.34 (1.85, 2.89)	21.67	7 (0.7%)	0 (0%)
ICAM1	1043	398230.84 (319427.14, 489731.46)	18.6 (18.29, 18.9)	7765.69	0 (0%)	0 (0%)
CD14	1043	1174615.6 (943288.8, 1475163.4)	20.16 (19.85, 20.49)	22460.76	0 (0%)	0 (0%)
CRP	976	1309806.4 (516764.28, 4048637.2)	20.32 (18.98, 21.95)	218860.69	0 (0%)	66 (6.3%)
IL1A	1	0.42 (0.42, 0.42)	-1.27 (-1.27, -1.27)	-0.02	1043 (99.9%)	0 (0%)
IL2	6	0.32 (0.32, 0.32)	-1.64 (-1.64, -1.64)	-0.12	1038 (99.4%)	0 (0%)
IL5	724	0.49 (0.03, 0.97)	-1.03 (-4.86, -0.04)	-0.78	320 (30.7%)	0 (0%)
INFBETA	8	3.08 (3.08, 3.08)	1.62 (1.62, 1.62)	6.44	1035 (99.2%)	0 (0%)

Table S2: Summary of inflammatory markers among EVD survivors.

	N uncensored measurements	Med. (Q1, Q3)	log2-transformed Med. (Q1, Q3)	Coefficient of Variation of log2-transformed variable	N (%) below LOD	N (%) above LOD
Macrophage-specific inflammatory markers						
MCP1	594	125.44 (93.84, 160.41)	6.97 (6.55, 7.33)	9.14	0 (0%)	0 (0%)
MCP2	593	21.51 (16.27, 30.23)	4.43 (4.02, 4.92)	3.82	1 (0.2%)	0 (0%)
TNFA	587	5.22 (4.12, 6.86)	2.39 (2.04, 2.78)	1.42	7 (1.2%)	0 (0%)
TNFR1	594	930.8 (785.92, 1097.36)	9.86 (9.62, 10.1)	29.78	0 (0%)	0 (0%)
TNFR2	594	2280.81 (1849.21, 2997.55)	11.16 (10.85, 11.55)	101.52	0 (0%)	0 (0%)
MIP1A	588	28.28 (20.7, 42.54)	4.82 (4.37, 5.41)	67.16	0 (0%)	1 (0.2%)
MIP1B	579	56.32 (42.68, 76.31)	5.82 (5.42, 6.25)	8.25	11 (1.9%)	0 (0%)
IL1B	100	0.1 (0.1, 0.1)	-3.25 (-3.25, -3.25)	-0.14	494 (83.2%)	0 (0%)
IL6	274	0.2 (0.2, 2.19)	-2.29 (-2.29, 1.13)	-7.44	320 (53.9%)	0 (0%)
MCSF	594	385.47 (293.34, 519.54)	8.59 (8.2, 9.02)	26.39	0 (0%)	0 (0%)
Non-macrophage-specific inflammatory markers						
VEGFA	592	44.78 (27.47, 87.53)	5.48 (4.78, 6.45)	20.29	2 (0.3%)	0 (0%)
VEGFB	594	40.89 (32.32, 55.14)	5.35 (5.01, 5.78)	9.59	0 (0%)	0 (0%)
GRANA	589	148.7 (115.91, 196.78)	7.22 (6.86, 7.62)	10.79	0 (0%)	0 (0%)
GRANB	588	12.99 (8.86, 20.82)	3.7 (3.15, 4.38)	32.42	1 (0.2%)	0 (0%)
IL10	593	5.33 (3.47, 11.37)	2.41 (1.79, 3.51)	6.33	1 (0.2%)	0 (0%)
IL2RA	594	1423.67 (1154.44, 1816.7)	10.48 (10.17, 10.83)	66.2	0 (0%)	0 (0%)
IL1RA	593	233.33 (178.21, 336.41)	7.87 (7.48, 8.39)	42.29	0 (0%)	0 (0%)
IL8	589	5.19 (3.77, 7.68)	2.37 (1.92, 2.94)	27.83	5 (0.8%)	0 (0%)
ICAM1	594	398165.74 (310585.3, 491424.32)	18.6 (18.24, 18.91)	8082.49	0 (0%)	0 (0%)
CD14	594	1201779 (973046.9, 1530234)	20.2 (19.89, 20.55)	23443.64	0 (0%)	0 (0%)
CRP	553	1393977.4 (543469.4, 4396932.1)	20.41 (19.05, 22.07)	220484.27	0 (0%)	40 (6.7%)
IL1A	1	0.42 (0.42, 0.42)	-1.27 (-1.27, -1.27)	-0.02	593 (99.8%)	0 (0%)
IL2	2	0.32 (0.32, 0.32)	-1.64 (-1.64, -1.64)	-0.14	592 (99.7%)	0 (0%)
IL5	396	0.45 (0.03, 0.94)	-1.15 (-4.86, -0.1)	-0.81	198 (33.3%)	0 (0%)
INFBETA	2	3.08 (3.08, 3.08)	1.62 (1.62, 1.62)	4.2	592 (99.7%)	0 (0%)

3

Table S3: Summary of inflammatory markers among EVD survivors with viral shedding.

	N uncensored measurements	Med. (Q1, Q3)	log2-transformed Med. (Q1, Q3)	Coefficient of Variation of log2-transformed variable	N (%) below LOD	N (%) above LOD
Macrophage-specific inflammatory markers						
MCP1	81	144.47 (119.42, 181.28)	7.17 (6.9, 7.5)	8.86	0 (0%)	0 (0%)
MCP2	81	24.47 (18.39, 36.91)	4.61 (4.2, 5.21)	2.78	0 (0%)	0 (0%)
TNFA	80	5.19 (4.23, 6.69)	2.38 (2.08, 2.74)	1.24	1 (1.2%)	0 (0%)
TNFR1	81	935.39 (770.18, 1121.73)	9.87 (9.59, 10.13)	29.4	0 (0%)	0 (0%)
TNFR2	81	2219.45 (1862.17, 3019.17)	11.12 (10.86, 11.56)	105.08	0 (0%)	0 (0%)
MIP1A	80	30.98 (24.52, 45.49)	4.95 (4.62, 5.51)	68.02	0 (0%)	0 (0%)
MIP1B	79	64.53 (48.09, 81.53)	6.01 (5.59, 6.35)	6.22	1 (1.2%)	0 (0%)
IL1B	18	0.1 (0.1, 0.1)	-3.25 (-3.25, -3.25)	-0.17	63 (77.8%)	0 (0%)
IL6	36	0.2 (0.2, 2.05)	-2.29 (-2.29, 1.04)	-11.45	45 (55.6%)	0 (0%)
MCSF	81	384.89 (302.14, 507.12)	8.59 (8.24, 8.99)	32.97	0 (0%)	0 (0%)
Non-macrophage-specific inflammatory markers						
VEGFA	81	68.82 (40, 178.54)	6.1 (5.32, 7.48)	20.06	0 (0%)	0 (0%)
VEGFB	81	38.62 (32.28, 51.15)	5.27 (5.01, 5.68)	9.25	0 (0%)	0 (0%)
GRANA	80	151.75 (115.87, 181.45)	7.25 (6.86, 7.5)	10.22	0 (0%)	0 (0%)
GRANB	80	17.23 (10.43, 29.71)	4.11 (3.38, 4.89)	15.4	0 (0%)	0 (0%)
IL10	81	4.82 (3.37, 9.43)	2.27 (1.75, 3.24)	6.63	0 (0%)	0 (0%)
IL2RA	81	1432.74 (1110.7, 1930.64)	10.48 (10.12, 10.91)	58.93	0 (0%)	0 (0%)
IL1RA	81	197.6 (162.14, 253.6)	7.63 (7.34, 7.99)	46.38	0 (0%)	0 (0%)
IL8	81	6.26 (5.1, 8.16)	2.65 (2.35, 3.03)	54.99	0 (0%)	0 (0%)
ICAM1	81	397635.87 (303755.93, 513478.48)	18.6 (18.21, 18.97)	8877.31	0 (0%)	0 (0%)
CD14	81	1200389 (1015965.5, 1616603)	20.2 (19.95, 20.62)	25834.41	0 (0%)	0 (0%)
CRP	76	860929.3 (461402.9, 2483080)	19.72 (18.82, 21.24)	201381.32	0 (0%)	5 (6.2%)
IL1A	0	0.42 (0.42, 0.42)	-1.27 (-1.27, -1.27)	0	81 (100%)	0 (0%)
IL2	0	0.32 (0.32, 0.32)	-1.64 (-1.64, -1.64)	0	81 (100%)	0 (0%)
IL5	53	0.51 (0.03, 0.94)	-0.97 (-4.86, -0.09)	-0.42	28 (34.6%)	0 (0%)
INFBETA	0	3.08 (3.08, 3.08)	1.62 (1.62, 1.62)	0	81 (100%)	0 (0%)

Table S4: Adjusted odds ratios by clinical finding for MIP-1B. The p-values shown here are not corrected for multiple comparisons. Odds ratios are adjusted for sex and age at enrollment (except for viral persistence which is adjusted for age and number of semen specimens collected) and refer to a two-fold increase in marker level.

	Survivors (N=594)		
		MIP1B	
	N (%)	Adj. OR (95% CI)	p-value
Urinary frequency	93 (15.7)	1.05 (0.8, 1.36)	0.74
Fatigue	112 (18.9)	1.08 (0.84, 1.39)	0.52
Headache	281 (47.3)	1.21 (0.99, 1.48)	0.062
Muscle pain	150 (25.3)	1 (0.8, 1.25)	0.98
Joint pain	325 (54.7)	0.84 (0.69, 1.04)	0.11
Memory loss	185 (31.1)	0.97 (0.79, 1.19)	0.78
Uveitis	207 (35)	0.96 (0.79, 1.18)	0.71
Acute uveitis	39 (6.6)	0.8 (0.57, 1.11)	0.17
Viral shedding	81 (33.3)	0.91 (0.61, 1.37)	0.66
Musculoskeletal abnormalities	47 (7.9)	1.18 (0.81, 1.71)	0.38
Neurological abnormalities	35 (5.9)	0.8 (0.56, 1.16)	0.24
Chest abnormalities	28 (4.7)	0.75 (0.49, 1.14)	0.17
Abdominal abnormalities	113 (19)	0.9 (0.71, 1.13)	0.36
Any of the findings	538 (90.6)	0.82 (0.56, 1.21)	0.31

5

Table S5: Adjusted odds ratios by finding for IL6. The p-values shown here are not adjusted for multiple comparisons. Odds ratios are adjusted for sex and age at enrollment (except for viral persistence which is adjusted for age and number of semen specimens collected) and refer to IL6 level being above (vs. below) the LOD.

	Survivors (N=594)			Survivors with viral shedding (N=81)		
		IL6			IL6	
	N (%)	Adj. OR (95% CI)	p-value	N (%)	Adj. OR (95% CI)	p-value
Urinary frequency	93 (15.7)	0.834 (0.52, 1.32)	0.44	93 (15.7)	1.389 (0.35, 5.65)	0.64
Fatigue	112 (18.9)	1.313 (0.86, 2.01)	0.21	112 (18.9)	0.498 (0.1, 2.2)	0.37
Headache	281 (47.3)	1.127 (0.8, 1.58)	0.49	281 (47.3)	0.838 (0.29, 2.34)	0.74
Muscle pain	150 (25.3)	1.198 (0.82, 1.76)	0.36	150 (25.3)	8.932 (1.99, 64.19)	0.0097
Joint pain	325 (54.7)	1.227 (0.87, 1.72)	0.24	325 (54.7)	2.272 (0.86, 6.29)	0.1
Memory loss	185 (31.1)	0.889 (0.62, 1.27)	0.52	185 (31.1)	1.29 (0.38, 4.31)	0.68
Uveitis	207 (35)	1.103 (0.78, 1.57)	0.58	207/591 (35)	1.382 (0.51, 3.73)	0.52
Acute uveitis	39 (6.6)	1.083 (0.55, 2.13)	0.81	39/591 (6.6)	7.401 (0.92, 157.02)	0.093
Viral shedding	81 (33.3)	1.218 (0.68, 2.19)	0.51	—	–	–
Musculoskeletal abnormalities	47 (7.9)	1.472 (0.79, 2.79)	0.23	47 (7.9)	1.411 (0.14, 13.77)	0.75
Neurological abnormalities	35 (5.9)	0.351 (0.15, 0.75)	0.01	35 (5.9)	0.226 (0.01, 1.7)	0.21
Chest abnormalities	28 (4.7)	2.237 (0.95, 5.68)	0.073	28 (4.7)	2.941 (0.29, 66.98)	0.39
Abdominal abnormalities	113 (19)	1.047 (0.68, 1.62)	0.84	113 (19)	0.863 (0.1, 6.23)	0.88
Any of the findings	538 (90.6)	1.975 (1.07, 3.82)	0.035	538 (90.6)	–	–

6
